# Phenotypic variability and identification of novel *YARS2* mutations in YARS2 mitochondrial myopathy, lactic acidosis and sideroblastic anaemia

**DOI:** 10.1186/1750-1172-8-193

**Published:** 2013-12-17

**Authors:** Lisa G Riley, Minal J Menezes, Joëlle Rudinger-Thirion, Rachael Duff, Pascale de Lonlay, Agnes Rotig, Michel C Tchan, Mark Davis, Sandra T Cooper, John Christodoulou

**Affiliations:** 1 Genetic Metabolic Disorders Research Unit, Kids Research Institute, Children’s Hospital at Westmead 2145, Sydney, Australia; 2 Discipline of Paediatrics & Child Health, University of Sydney 2006, Sydney, Australia; 3 Discipline of Genetic Medicine, Sydney Medical School, University of Sydney 2006, Sydney, Australia; 4 Architecture et Réactivité de l’ARN, Université de Strasbourg, CNRS, IBMC, Strasbourg 67084, France; 5 Centre for Medical Research, University of Western Australia and Western Australian Institute for Medical Research, Perth, Western Australia 6009, Australia; 6 Université Paris Descartes and INSERM U781, Hôpital Necker-Enfants Malades, Paris 75015, France; 7 Department of Genetic Medicine, Westmead Hospital, Sydney, Australia; 8 Diagnostic Genomics, PathWest Laboratory Medicine, QEII Medical Centre, Nedlands, WA 6009, Australia; 9 Institute for Neuroscience and Muscle Research, Kids Research Institute, Children’s Hospital at Westmead 2145, Sydney, Australia; 10 Western Sydney Genetics Program, Children's Hospital at Westmead, Locked Bag 4001, Westmead, New South Wales 2145, Australia

**Keywords:** Mitochondrial respiratory chain, Myopathy with lactic acidosis and sideroblastic anemia, Aminoacyl tRNA-synthetase, Mitochondrial myopathies, Muscle diseases, Lactic acidaemia, Inborn error of metabolism, Mutation

## Abstract

**Background:**

Mutations in the mitochondrial tyrosyl-tRNA synthetase (*YARS2*) gene have previously been identified as a cause of the tissue specific mitochondrial respiratory chain (RC) disorder, Myopathy, Lactic Acidosis, Sideroblastic Anaemia (MLASA). In this study, a cohort of patients with a mitochondrial RC disorder for who anaemia was a feature, were screened for mutations in *YARS2*.

**Methods:**

Twelve patients were screened for *YARS2* mutations by Sanger sequencing. Clinical data were compared. Functional assays were performed to confirm the pathogenicity of the novel mutations and to investigate tissue specific effects.

**Results:**

Pathogenic*YARS2* mutations were identified in three of twelve patients screened. Two patients were found to be homozygous for the previously reported p.Phe52Leu mutation, one severely and one mildly affected. These patients had different mtDNA haplogroups which may contribute to the observed phenotypic variability. A mildly affected patient was a compound heterozygote for two novel *YARS2* mutations, p.Gly191Asp and p.Arg360X. The p.Gly191Asp mutation resulted in a 38-fold loss in YARS2 catalytic efficiency and the p.Arg360X mutation did not produce a stable protein. The p.Phe52Leu and p.Gly191Asp/p.Arg360X mutations resulted in more severe RC deficiency of complexes I, III and IV in muscle cells compared to fibroblasts, but had relatively normal YARS2 protein levels. The muscle-specific RC deficiency can be related to the increased requirement for RC complexes in muscle. There was also a failure of mtDNA proliferation upon myogenesis in patient cells which may compound the RC defect. Patient muscle had increased levels of PGC1-α and TFAM suggesting mitochondrial biogenesis was activated as a potential compensatory mechanism.

**Conclusion:**

In this study we have identified novel *YARS2* mutations and noted marked phenotypic variability among YARS2 MLASA patients, with phenotypes ranging from mild to lethal, and we suggest that the background mtDNA haplotype may be contributing to the phenotypic variability. These findings have implications for diagnosis and prognostication of the MLASA and related phenotypes.

## Background

Mitochondrial respiratory chain (RC) disorders are among the most prevalent inborn errors of metabolism with an incidence of ~1:8000 births [1]. They are a clinically heterogeneous group of disorders caused by mutations in either mitochondrial or nuclear DNA [2]. Mitochondrial RC function requires the coordinated expression of both mitochondrial and nuclear encoded genes, to supply the majority of cellular energy demands. The respiratory capacity is regulated depending on the energy requirements of the tissue and prevailing metabolic conditions [3]. Compensatory mechanisms, such as mitochondrial proliferation, may be activated in cases of mitochondrial RC dysfunction [4]. The dynamic nature of the mitochondrial RC may be one of the contributing factors to the tissue-specific manifestations, and inter- and intra-familial phenotypic variability that is frequently observed in mitochondrial RC disorders. These factors further complicate diagnosis and prognostication [5].

Many of the mitochondrial RC disorders result from genetic defects that impair mitochondrial protein synthesis, with mutations reported in mitochondrial tRNAs, aminoacyl-tRNA synthetases, elongation factors and ribosomal proteins [6]. Curiously, these do not result in the same phenotype, but a spectrum of disorders. Even mutations within the mitochondrial aminoacyl-tRNA synthetase (ARS2) family of proteins, which are responsible for connecting mitochondrially located tRNAs with their cognate amino acids, result in different clinical phenotypes with discrete tissue-specific involvement. *DARS2*[7], *EARS2*[8] and *MARS2*[9] mutations cause leukoencephalopathy; each with other gene-specific CNS involvement; *RARS2* mutations result in pontocerebellar hypoplasia [10]; *HARS2* and *LARS2* mutations result in ovarian dysgenesis and sensorineural hearing loss [11,12]; *AARS2* mutations cause infantile cardiomyopathy [13]; *SARS2* mutations cause hyperuricemia, pulmonary hypertension, renal failure in infancy and alkalosis [14]; *FARS2* mutations cause mitochondrial encephalopathy [15]; and we recently identified a *YARS2* mutation as a novel cause of a mitochondrial RC disorder characterised by a skeletal myopathy, lactic acidosis and sideroblastic anaemia (MLASA) [16]. MLASA has also been associated with mutations in *PUS1 *[17]. The reason for the diverse range of symptoms resulting from mutations in mitochondrial aminoacyl-tRNA synthetases is unclear, however several cytoplasmic aminoacyl-tRNA synthetases have been found to have additional functions besides their role in protein synthesis, and it may be that other functions of mitochondrial aminoacyl-tRNA synthetases influence the distinct pathogenesis of aminoacyl-tRNA synthetase disorders [18]. The tissues affected by mitochondrial RC disorders are commonly those with higher energy requirements and/or those with greater sensitivity to mitochondrial dysfunction, which cannot be functionally sustained in the face of compromised mitochondrial RC output. However, this does not explain why different patients may manifest abnormalities of brain, heart or skeletal muscle function for example, but not all three of these energy-demanding tissues. It is clear that the pathogenesis of mitochondrial RC disorders is more complicated than the simple concept of metabolic demands, and a better understanding of the basis for tissue-specific manifestations and phenotypic variability is needed.

In this study we further define the clinical spectrum associated with *YARS2* mutations identified in three probands. We identified the p.Phe52Leu mutation, originally described as a likely founder mutation [16], in two other families. Considerable phenotypic variability was observed among the p.Phe52Leu YARS2 patients, which has important implications for diagnosis. We propose that this phenotypic variability may in part be due to differences in mtDNA haplogroups among patients. In addition, we found two novel *YARS2* mutations in a patient and defined their biochemical characteristics and tRNA^Tyr^ aminoacylation dysfunction. We also examined the basis for the tissue-specific manifestation of YARS2 mitochondrial myopathy.

## Methods

### Clinical information

The Human Research Ethics Committee of the Children’s Hospital at Westmead approved this research.

A cohort of 12 patients was selected for *YARS2* screening. Selection criteria included presence of anaemia, with or without lactic acidosis (10/12 patients had lactic acidosis), with or without skeletal myopathy (6/12 patients displayed myopathy/hypotonia), and a demonstrated respiratory chain enzyme deficiency. Nine of the 12 patients screened were of French origin, one was Italian and the other two were Australians of Lebanese origin, unrelated to the two original families for which a *YARS2* mutation was previously described [16]. Clinical histories for patients for whom we identified pathogenic *YARS2* mutations are given below (patients 4, 5 & 6) and previously reported cases are reviewed (patients 1, 2 & 3). Clinical features of the other patients are presented in Additional file 1.

Patient 1 and 2 are siblings of consanguineous parents and have been described previously [16]. Briefly, within the first 3 months of life P1 developed transfusion dependent sideroblastic anaemia and had persistent lactic acidaemia and hypertrophic cardiomyopathy. The cardiomyopathy spontaneously resolved. He had progressive exercise tolerance and at 17 years he developed dysphagia and acute respiratory difficulties and succumbed at 18 years of age. P2 also developed transfusion dependent sideroblastic anaemia in infancy and had persistent lactic acidaemia and progressive muscle weakness, similar to P1. At 15 years she developed dysphagia and had a gastrostomy inserted. Remarkably, at 17 years of age P2 showed improved muscle strength and stamina, and no longer required blood transfusions (previously being given every six weeks). She is currently 20 years old. Patient 3 [16], born to consanguineous parents, had delayed motor milestones and developed sideroblastic anaemia at 7 years, as well as lactic acidaemia. Her parents refused to allow her to have blood transfusions and she was treated with complementary therapies. At 24 years she had a mild skeletal myopathy, anaemia and mild peripheral muscle weakness.

Patient 4, the second child of non-consanguineous parents of Lebanese origin, is not known to be related to the other patients described here. There were no antenatal concerns. She was born at term by normal delivery with good Apgar scores. She had low birth weight of 2.7 kg but there were no immediate postnatal problems. She fed well by formula and was gaining weight at home.

There is a family history of a maternal aunt with sickle cell anaemia. The parents have a healthy 3-year old daughter, and there is a history of a prior fetal demise at 12 weeks gestation.

The proband presented at 8 weeks of age in acute hypotensive shock needing cardiopulmonary resuscitation following a few days of rhinorrhoea, loose stools and associated lethargy and feeding difficulties. A venous blood gas at the time of arrest showed pH 6.56, bicarbonate of 4.2 mmol/L, BE -28.8, anion gap of 30 and lactate of 27 mmol/L (normal range 0.7 – 2.0). A CT scan of the brain was normal. Urine metabolic screening showed increased lactate and ketones. Plasma amino acids, triglycerides, free fatty acids, uric acid, blood ammonium and serum transferrin isoforms were normal. There was a slightly low plasma total carnitine level noted.

She had associated multisystem problems. These included significant concentric left ventricular hypertrophy with altered echotexture, and abnormal diastolic function. She had an initial mild pericardial effusion which later required pericardial drainage. She also had hepatomegaly with raised transaminases and coagulopathy. She had possible seizures, managed with anticonvulsants. A subsequent MRI/MRS of the brain showed cerebral atrophy with normal spectroscopy and no lactate peak.

She had anaemia, with the lowest haemoglobin being 42 g/L, and required intermittent packed cell transfusions. Bone marrow aspirate showed reduced erythropoiesis with prominent red cell vacuolation in the red cell precursors and ringed sideroblasts. There was no evidence of a storage disorder on bone marrow biopsy.

She did not tolerate weaning from mechanical ventilation. Impaired cardiac output in conjunction with the above associated issues led to eventual decision for withdrawal of active intensive care. She succumbed at three months of age.

Subsequent to identification of the p.Phe52Leu YARS2 mutation, they have had two pregnancies which underwent prenatal testing, and were then terminated as both were found to be homozygous for the mutation.

Patient 5 is one of four siblings to non-consanguineous Lebanese parents (although they are from the same village in Northern Lebanon). Her sister was diagnosed with sideroblastic anaemia in Lebanon in her mid 20s and had been transfusion dependent for around 10 years. She ultimately died of cirrhosis induced by iron overload 10 years after commencing blood transfusions. This individual was reported to be an otherwise fairly healthy young woman without any other medical concerns.

Patient 5 was noted to be anaemic at the age of 23. An initial bone marrow performed in Lebanon reportedly showed a form of refractory anaemia for which she had four erythropoietin injections. Subsequently, she returned to Australia and a second bone marrow aspirate four years later demonstrated ringed sideroblasts. At that time, her lactate was elevated at 3.24 mmol/L with a pyruvate of 0.13 mmol/L. On review a year later, her lactate was 5.10 mmol/L and haemoglobin was low at 104 g/L. Creatine kinase was 30 U/L, at the lower end of the normal range.

Patient 5 was found to have scoliosis at the age of 11 years, and she went on to have titanium rods inserted which were subsequently removed secondary to infection. She has subsequently had a spinal fusion. She also has endometriosis.

Her 6-minute walk time was reduced at 353 m. Her pulmonary function tests demonstrated a mild restrictive defect with the FVC of 70% predicted and FEV1 of 70% predicted; the Diffusing lung capacity for carbon monoxide (DLCO) was normal. Her ECG and echocardiography were also normal.

On examination, there was no organomegaly, and her cardiovascular and respiratory examination was normal. She had a long, well-healed scar over her spine, with a slight residual right thoracic scoliosis. She had subtle bilateral hip flexor weakness, but her power otherwise was intact. Deep tendon reflexes and sensation were normal. There were no problems with gait or balance. Intelligence was normal.

Patient 6, the child of non-consanguineous parents of French origin required blood transfusion for sideroblastic anaemia in the first year of life, which resolved spontaneously. A bone marrow examination during the first year of life showed the presence of megakaryocytes, signs of erythroblastic dysplasia, and an excess of sideroblasts. A partial deficiency in complex IV was detected in lymphocytes (muscle biopsies were not performed), and abnormal assembly of RC complexes I, III, IV and V were observed on Blue Native-PAGE of fibroblasts. At 3 years of age there was a discrete thickening of the myocardium, which normalised by age 5 years. No skeletal myopathy was present, but she had nystagmus and strabismus. Liver function tests were normal. Mild but permanent lactic acidaemia (3 mmol/L) was found at 5 years. Lactic acidaemia was also present at 6 years and she reported fatigue on prolonged walking or climbing stairs, together with leg pain and muscle weakness. There was no evidence of anaemia. Intelligence was normal.

### *YARS2, PUS1* and mtDNA sequencing

All *YARS2* and *PUS1* coding exons were PCR amplified from gDNA and Sanger DNA sequencing performed by Macrogen (Korea). mtDNA from p.Phe52Leu YARS2 patients was sequenced at the Lotterywest State Biomedical Facility Genomics, Royal Perth Hospital. mtDNA haplogroups were assigned using Haplofind [19].

### Cloning and aminoacylation assays

Recombinant YARS2 mutant proteins were generated to assess their tyrosylation activity. Recombinant wild-type and YARS2 variants were cloned, expressed and purified from *E. coli* as previously described [20]. *In vitro* tRNA^Tyr^ aminoacylation assays were performed using previously described methods [20]. Apparent kinetic parameters were determined from Lineweaver-Burk plots in the presence of 5 to 27 nM YARS2 (depending on the variant) and native *E. coli* tRNA^Tyr^ (Sigma), with concentrations ranging from 0.28 to 2.1 μM. Experimental errors for k_cat_ and K_*m*_ varied at most by 20%. Numerical values are averages of at least two independent experiments.

### Native and Myo-D forced myogenesis

Primary patient and control fibroblast cell lines were routinely cultured in DMEM: F12 (Life Sciences) containing 20% Amniomax (Life Sciences) and 10% heat inactivated fetal bovine serum (FBS; In Vitro Technologies), penicillin (50 μg/ml) and streptomycin (50 μg/ml; Life Sciences) and primary myoblast cell lines were cultured in DMEM: F12, 20% Amniomax, 20% FBS, in a humidified incubator containing 5% CO_2_ and maintained at 37°C. Fibroblasts were transduced and differentiated using a lentiviral MyoD vector as previously described [21], except that cells were seeded onto plates coated with 0.15 mg/ml rat tail collagen (BD Biosciences). Myoblasts were differentiated in the same media as described for fibroblast transdifferentiation. Cells were harvested at day 6 of differentiation for enzyme assays, immunoblotting or DNA extraction.

### RC enzyme assays

Respiratory chain enzyme activities were determined as previously described [22].

### Immunoblotting

Immunoblotting was as previously described [16], with the following modifications. Membranes were probed with 1:500 anti-OXPHOS (Abcam) for 2 h at room temperature or with 1:500 anti-YARS2 (N-terminal, Abgent), 1:10000 anti-porin (Abcam), 1:1000 anti-mitofilin (Abcam), 1:500000 anti-α-actinin-2 (a generous gift to the INMR from Prof Alan Beggs, Harvard Medical School, Boston Children’s Hospital, Division of Genetics), 1:500 anti-PGC1-α (Sigma) or 1:500 anti-TFAM (Abcam) overnight at 4°C. Densitometry was performed as previously described [16] and protein levels were normalised to porin protein levels.

### DNA extraction

DNA was isolated from patient fibroblast cell pellets and skeletal muscle tissues and sex- and age-matched controls using a QIAamp DNA mini kit (QIAGEN). Tissues were homogenized using a TissueRuptor (QIAGEN). Samples were treated with RNase A (QIAGEN).

### mtDNA/nDNA determination by qPCR

A mitochondrial encoded gene (*ND1*) was quantified relative to a single copy number nuclear encoded gene (*β2M*) [23] to determine the mtDNA content in patient and control myoblasts, myotubes and muscle. These genes were amplified in separate reactions containing 40 ng total DNA, 1 Unit of Immolase™ DNA Polymerase (Bioline), and a final concentration of 1.5 mM MgCl_2_, 500 pM of each primer, 2% DMSO and 1 M betaine. Standard curves were generated by amplifying known amounts of a pTOPO2.1/B2M or pTOPO2.1/ND1 plasmid. The cycle conditions were: 95°C for 12 minutes, (95°C for 15 s, 60°C for 15 s, 72°C for 20 s) × 35 cycles. Primer sequences were (5' to 3'): ND1_F – CATAAAACTCTTCACCAAAGAGCC; ND1_R – GGGGTTCATAGTAGAAGAGCGA; β2M_F – TGCTGTCTCCATGTTTGATGTATCT; β2M_R – TCTCTGCTCCCCACCTCTAAGT.

### Statistical analyses

Generalised linear models for univariate analysis (SPSS v.19, IBM) were used to assess the effect of cell type (fibroblast, forced myotube), sample (patient P2, patient P6, control) and their interaction with normalised Complex IV activity and normalised Complex IV/CS activity. Generalised linear models for univariate analysis were also used to assess the effect of cell type (myoblast, myotube), sample (patient P2, control) and their interaction with relative mtDNA/nDNA. Where there was a significant interaction between cell type and sample, effects were analysed independently. No adjustments were made for multiple statistical analyses. An independent sample t-test (SPSS v.19, IBM) was used to test for differences in relative mtDNA/nDNA between patient and control muscle.

## Results

### Additional p.Phe52Leu YARS2 patients display marked clinical variability

We have previously identified a p.Phe52Leu *YARS2* mutation in three MLASA patients (P1, P2 and P3; Table 1) who demonstrated a muscle-specific RC deficiency affecting complexes I, III and IV [16]. Screening of two additional unrelated patients with similar clinical features identified Patients P4 and P5 as also being homozygous for the missense mutation c.156C > G (p.Phe52Leu). The non-consanguineous parents of P4 showed both were heterozygous for the c.156C > G substitution. Parental DNA was not available for P5.

**Table 1:** Clinical data for YARS2 patients

**Patient**	**P1**		**P2**^***a***^		**P3**		**P4**		**P5**		**P6**	
**YARS2 variant**	[c.156C > G]; [c.156C > G] [p.Phe52Leu]; [p.Phe52Leu]		[c.156C > G]; [c.156C > G] [p.Phe52Leu]; [p.Phe52Leu]		[c.156C > G]; [c.156C > G] [p.Phe52Leu]; [p.Phe52Leu]		[c.156C > G]; [c.156C > G] [p.Phe52Leu]; [p.Phe52Leu]		[c.156C > G]; [c.156C > G] [p.Phe52Leu]; [p.Phe52Leu]		[c.572G > A]; [c.1078C > T] [p.Gly191Asp]; [p.Arg360X]	
**mtDNA haplogroup**	K1a4f1		K1		K1a4f		T1a8		H		ND	
RC Enzyme Activity^*b*^	Muscle	Fibroblast	Muscle	Fibroblast	Muscle	Fibroblast	Muscle	Liver	Muscle	Fibroblast	Muscle	Lymphocyte
**Complex I/CS**	8	62	3	ND	25	80	2	49	ND	ND	ND	85
**Complex II/CS**	150	116	139	ND	110	39	132	191	ND	ND	ND	94
**Complex III/CS**	ND	107	3	ND	ND	56	4	105	ND	ND	ND	67
**Complex IV/CS**	12	89	2	ND	7	52	3	85	ND	ND	ND	70
**CS**^***c***^	236	67	319	ND	94	94	376	254	ND	ND	ND	319
**Sideroblastic anaemia**	Severe		Moderate		Mild		Severe		Mild		Mild	
	Transfusion dependent from birth		Transfusion dependent from birth to 17 y		From 7 y		Transfusion dependent from birth		From 23 y		Transfusion dependent during first year	
					Not transfused for personal reasons				Not transfusion dependent			
					Hb 70–110 g/L		Hb 42 g/L		Hb 104 g/L			
**Lactic acidosis**	Severe		Moderate		Mild		Severe		Mild		Mild	
	3–13.7 mmol/L		2.5-8.4 mmol/L		4.1 mmol/L		27 mmol/L		3.24-5.10 mmol/L		2–3 mmol/L	
**Myopathy**	Severe		Moderate		Mild		ND		Mild		Mild	
**Other features**	Cardiomyopathy in infancy		Currently 20 y with improvement in clinical features		Currently 27 y		Cardiomyopathy		Currently 28 y		Currently 6 y	
	Deceased at 18 y						Deceased at 3 mo					

^*a*^Sibling of P1.

^*b*^Values expressed as % residual activity.

^*c*^Citrate synthase activity was expressed relative to protein.

CS = citrate synthase.

ND = not determined.

Despite similar RC enzyme results in muscle, clinical severity varied greatly among patients (Table 1). Patient P4 showed a severe phenotype and died at 3 months due to hypertrophic cardiomyopathy. P1 also had cardiomyopathy in infancy, however this resolved without specific therapy. P5 did not develop symptoms until adulthood, characterised by mild myopathy and transfusion-independent anaemia. Patient P2 presented at birth with a moderate myopathy and transfusion-dependent anaemia. Remarkably, at 17 years of age her clinical condition spontaneously improved, while her sibling, P1, had progressive muscle weakness, becoming wheelchair bound by 14 years and died of respiratory failure at 18 years of age. The four older *YARS2* p.Phe52Leu patients all had normal cognitive function. All five of the homozygous p.Phe52Leu YARS2 patients are Australians of Lebanese origin consistent with our proposal that p.Phe52Leu represents a rare founder mutation [16].

mtDNA sequencing revealed that although the patients were of the same ethnicity, they did not all have the same mtDNA haplogroup (Table 1; see Additional file 2 for a complete list of mtDNA variations). The 3 previously reported patients (P1,P2 & P3) all belonged to haplogroup K. P4, the most severely affected patient, belonged to haplogroup T and P5, the least severely affected patient belonged to haplogroup H.

### *YARS2* screening identifies new mutations

*YARS2* and *PUS1* mutation screening was performed on a cohort of ten French or Italian patients with demonstrated RC enzyme deficiency who also presented with anaemia and at least one of the other MLASA features, ie. lactic acidosis or myopathy. One patient, P6 (Table 1) had two novel *YARS2* mutations, a heterozygous missense mutation c.572G > A (p.Gly191Asp) and a heterozygous nonsense mutation c.1078C > T (p.Arg360X). Patient P6 *YARS2* variations were not present in dbSNP (NCBI), 1000 genomes (browser.1000genomes.org), or the ESP database (evs.gs.washington.edu). Screening of parental DNA showed that each parent carried one or the other of the mutations.

Patient P6 ([p.Gly191Asp];[p.Arg360X]) has mild lactic acidosis and myopathy, and only required blood transfusions during the first year of life (Table 1). Impaired complex IV enzymology was demonstrated via RC testing of lymphocytes and defective assembly of complexes I, III, IV & V on Blue Native-PAGE of fibroblast samples (data not shown).

*YARS2* variants were identified in two other patients (P7 & P8) however they are unlikely to be pathogenic. These are described in Additional file 1. No *PUS1* mutations were identified in this cohort of patients.

### p.Gly191Asp YARS2 displays reduced aminoacylation activity

To determine the functional impact of the p.Gly191Asp YARS2 mutation on tyrosylation capacity, *in vitro* assays were performed using *E. coli* tRNA^Tyr^ as substrate. Gly191 lies within the catalytic domain of YARS2 [24], and the protein prediction program SIFT (http://sift.jcvi.org/) predicts the Gly191Asp variation to be damaging.

The aminoacylation assay measured incorporation of [^14^C]-tyrosine on native *E. coli* tRNA^Tyr^ by recombinant YARS2. The p.Gly191Asp mutation did not significantly affect the K_*m*_ of the reaction (Table 2), indicating that YARS2 interaction with tRNA^Tyr^ was apparently unaffected. However the catalytic rate (k_cat_) was perturbed, resulting in reduced tyrosylation activity. The p.Gly191Asp variant resulted in a 38-fold decrease in catalytic efficiency (k_cat_ / K_*m*_) as compared to the wild-type enzyme, a greater reduction than the previously reported p.Phe52Leu mutation (9-fold decrease) [16]. This result was consistent with the SIFT prediction of the severity of the p.Gly191Asp variation on protein function.

**Table 2:** **Kinetic parameters for tyrosylation of tRNA**^**Tyr **^**by wild-type and p.Gly191Asp YARS2 recombinant proteins**

**YARS2 Variant**	**K**_***m ***_**(μM)**	**k**_**cat **_**(min**^**-1**^**)**	**k**_**cat**_**/K**_***m ***_**(Efficiency)**	**Loss of Efficiency**^***a ***^**(Fold change)**
WT	1	34	34	1
p.Gly191Asp	1	0.9	0.9	38

^*a*^Loss of efficiency is calculated relative to the wild-type YARS2.

### YARS2 protein levels are unaffected while RC complex expression levels are altered

**Figure 1:**
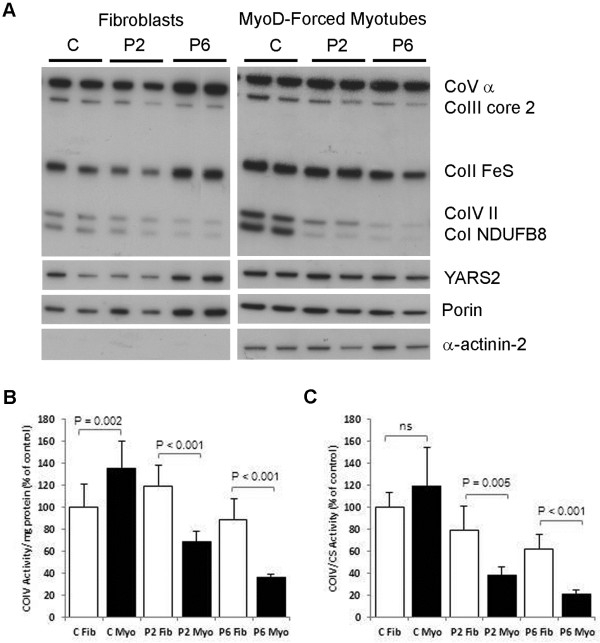
**RC complex deficiency is more severe in muscle cells.** Immunoblot of RC complex and YARS2 levels in YARS2 patient fibroblasts and MyoD-forced myotubes **(A)**. C = control, P2 = [p.Phe52Leu];[p.Phe52Leu] YARS2, P6 = [p.Gly191Asp];[p.Arg360X] YARS2. Complex IV activity was measured relative to total protein **(B)** and citrate synthase activity **(C)** in control (C) and patients (P2 & P6) fibroblasts (Fib) and MyoD-forced myotubes (Myo). Experiments were performed at least twice, in triplicate each time. Control fibroblasts were normalised to 100%. Values are the mean ± SD.

The expression level of YARS2 and the RC complexes was investigated by immunoblotting of patient P6 fibroblast and myotubes (Figure 1a). Due to the muscle-specific nature of the YARS2 RC deficiency and the unavailability of P6 muscle samples, myotubes were generated by transdifferentiation of fibroblasts using a lentiviral vector expressing MyoD [21], a master regulatory myogenic transcription factor. This model was validated by demonstrating a similar induction of mitochondrial RC expression levels via native and Myo-D forced myogenesis for patient P2, for whom a myoblast cell line was available (Additional file 3). Protein levels of YARS2 were not significantly altered in either patient P6 fibroblasts or MyoD-forced myotubes, relative to controls, suggesting that reduced YARS2 protein levels are not the cause of the muscle-specific phenotype. This was also the case for p.Phe52Leu YARS2 (P2), which is shown for comparison (Figure 1a). There was no evidence of a truncated YARS2 protein that might be expected to arise from the p.Arg360X mutation in P6. Sequencing of P6 cDNA showed that the c.1078C > T (p.Arg360X) mutant transcript was unstable and qPCR showed that total YARS2 mRNA levels were ~50% of controls (Additional file 4). Porin (VDAC), an outer mitochondrial membrane protein, and α-actinin-2, a muscle specific protein, were used as loading controls, and to demonstrate the efficacy of the transdifferentiation of fibroblasts into myotubes (Figure 1a). Immunoblotting of patient fibroblast samples showed RC complex I & IV levels were 50-60% of control levels (Figure 1a). The observed complex I & IV deficiencies were more severe in MyoD-forced myotubes (P2:25-50% of control levels; P6:5-10% of control levels) compared to fibroblasts, consistent with a muscle-specific phenotype.

### RC deficiency is more severe in muscle cells

We measured complex IV activity in myotubes derived from MyoD-forced myogenesis of fibroblasts from *YARS2* patients P2 (p.Phe52Leu) and P6 ([p.Gly191Asp];[p.Arg360X) to confirm that the reduced RC complex levels (Figure 1a) correlated with a reduction in RC enzyme activity. Transdifferentiation of fibroblasts to myotubes resulted in a significant increase in complex IV activity relative to total protein in normal controls (P = 0.002; Figure 1b), consistent with the increased relative levels of mitochondrial RC subunits detected by Western blot (Figure 1a and Additional file 3). In contrast, for both the patient P2 & P6 cell lines there was a decrease in complex IV activity relative to total protein (P < 0.001; Figure 1b) and relative to citrate synthase (P ≤ 0.005; Figure 1c). Both patient P2 fibroblasts and patient P6 fibroblasts had significantly lower complex IV/CS activity compared to control fibroblasts (P = 0.02 and P < 0.001 respectively; Figure 1c). However, MyoD-forced myogenesis revealed a deficit in Complex IV/CS activity that was < 50% of control levels and could be considered diagnostic. These results were consistent with the more severely reduced levels of Complex IV observed in patient myotubes compared to fibroblasts by Western blot (Figure 1a).

**Figure 2:**
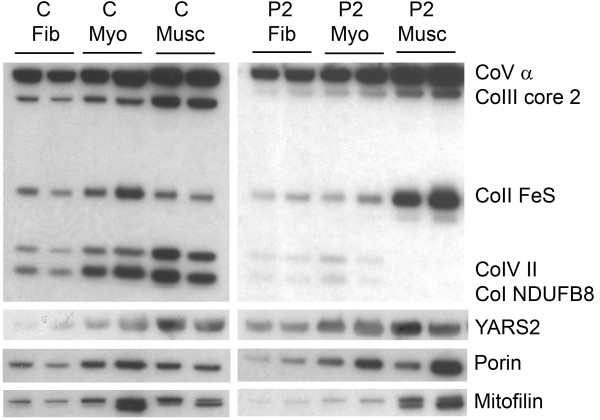
**RC complex levels are dysregulated on myogenesis in patient muscle cells.** Control (C) MyoD-forced myotubes (Myo) and muscle (Musc) have higher RC complex levels than fibroblasts (Fib) while patient (P2) MyoD-forced myotubes and muscle are deficient in Complex I (CoI NDUFB8), and IV (CoIV II).

The muscle-specific phenotype of YARS2 patients may be related to the increased requirements for RC complexes in muscle [25,26]. Thus, we compared the relative levels of mitochondrial RC expression levels in 10 μg of total protein from fibroblasts, MyoD-forced myotubes, and skeletal muscle (Figure 2). In control samples, there was a ~2-fold increase in the relative levels of all RC complexes with transdifferentiation from fibroblasts to myotubes, and a further ~2-fold incremental increase of RC expression levels between myotubes and skeletal myofibres (Figure 2). However, cells from Patient 2 failed to induce mitochondrial RC complex I and IV expression with myogenesis, either via forced myogenesis of fibroblasts (Figure 2) or indeed native myogenesis of myoblasts (Additional file 3). Skeletal muscle samples from Patient 2 demonstrated a severe deficiency of complex I and IV, with compensatory upregulation of complex II. Thus, our results suggest that patients with *YARS2* mutations can generate sufficient RC complex I, and IV in fibroblasts, but not in muscle where there is increased demand.

There were higher levels of mitofilin and porin (VDAC) in control myotubes and muscle compared to fibroblasts, indicative of greater numbers (or size) of mitochondria with myogenesis (Figure 2); consistent with increased RC complex levels. Skeletal muscle from patient P2 showed higher levels of mitofilin and porin compared to control muscle, suggesting mitochondrial proliferation was upregulated in response to the RC deficiency. There was no evidence of a compensatory upregulation of YARS2 in patient muscle.

### Mitochondrial biogenesis is altered in patient muscle cells

**Figure 3:**
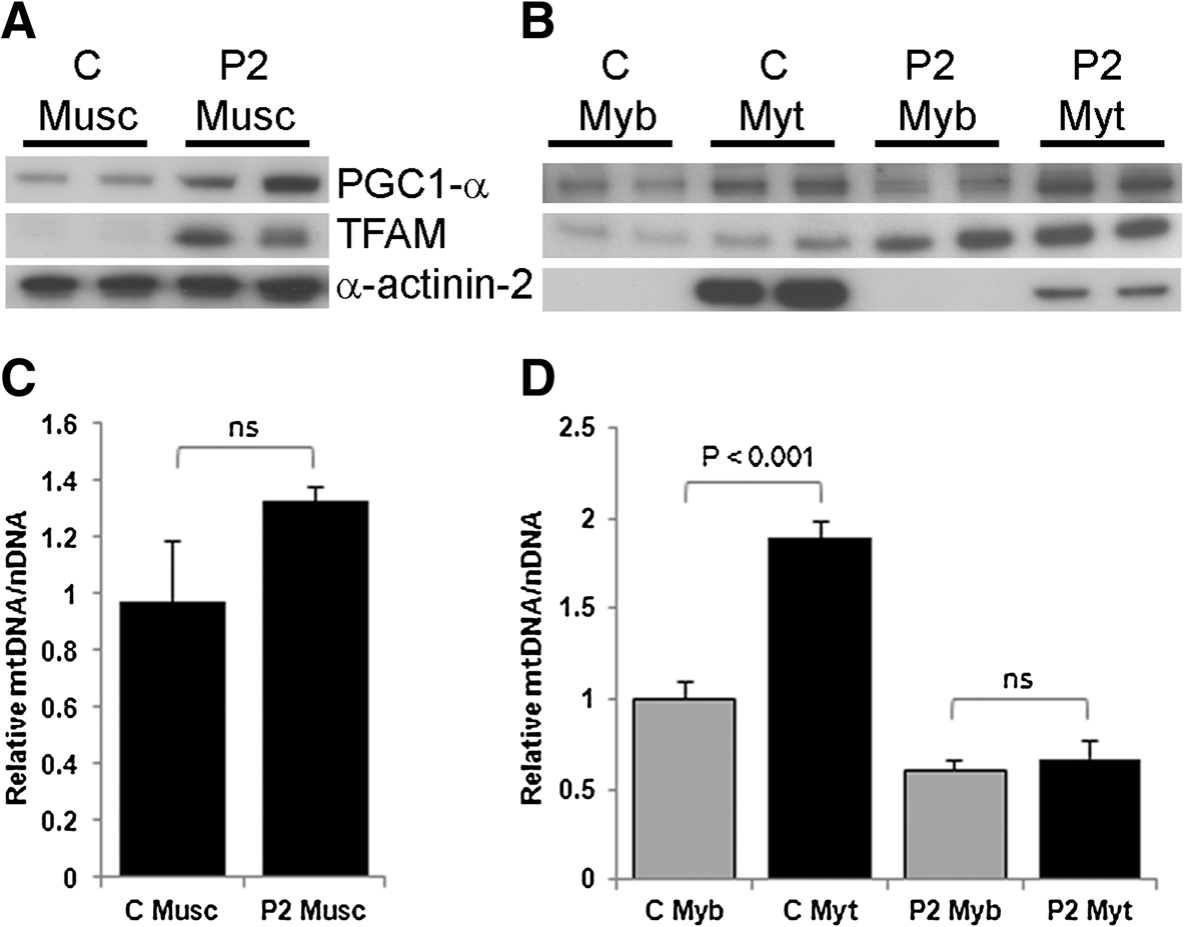
**Mitochondrial biogenesis is altered in patient muscle cells.** Immunoblot of RC complex, PGC1-α, and TFAM levels in control **(C)** and patient P2 muscle (Musc) **(A)**, myoblasts (Myb) and myotubes (Myt) **(B)**. α-actinin-2 levels are shown as a loading control for muscle and as an indicator of myogenesis in myotubes. Mitochondrial DNA copy number was determined by qPCR in control (C) and patient (P2) muscle **(C)** and myoblasts and myotubes **(D)**. Experiments were performed twice, in triplicate each time. Results are expressed relative to a control muscle **(C)** or myoblast **(D)** value of 1. Values are the mean ± SD. ns = not significantly different.

We further investigated the role of mitochondrial proliferation in YARS2 pathology. PGC1-α is a transcription co-activator which promotes mitochondrial biogenesis [27] and TFAM controls mtDNA copy number and transcription [28]. Elevated levels of PGC1-α and TFAM were seen in patient P2 muscle compared to controls (Figure 3a), consistent with induction of mitochondrial proliferation pathways. Interestingly, relative levels of mtDNA/nDNA were not significantly higher in patient P2 muscle compared to control muscle (P = 0.09; Figure 3c), despite our biochemical evidence showing higher relative levels of mitochondrial structural proteins and induction of PGC1-α and TFAM pathways.

We next assessed the relative levels of PGC1-α and TFAM with myogenesis in patient and control myoblasts. PGC1-α levels were similar in patient and control myoblasts and myotubes, and showed ~2-fold upregulation with myogenesis (Figure 3b). Levels of TFAM also increased incrementally with myogenesis, but showed 3-fold higher expression levels in patient myoblasts and myotubes, relative to controls (Figure 3b). Analysis of the relative levels of mtDNA/nDNA during myogenesis showed there was strong evidence of an interaction between cell type and sample (P < 0.001) so cell types were compared within samples. Myogenesis of control myoblasts induced a ~2-fold increase in the relative levels of mtDNA/nDNA (P < 0.001). However, myogenesis-induced upregulation of mtDNA proliferation was not observed for P2 (P = 0.22; Figure 3d). The elevated level of TFAM observed in patient muscle tissue and cells, does not appear to result in elevated mtDNA levels.

## Discussion

Here we refine the clinical spectrum associated with *YARS2* mutations through mutation screening of a cohort of mitochondrial RC disorder patients with anaemia. Five patients from four families homozygous for the p.Phe52Leu substitution are now identified, revealing marked clinical variability in age of onset and severity of sideroblastic anaemia and myopathy. We hypothesize that the mtDNA background may contribute to the observed clinical variability among patients. P4, the most severely affected patient who died in infancy due to hypertrophic cardiomyopathy, belonged to haplogroup T. Haplogroup T has been associated with risk of developing hypertrophic cardiomyopathy [29], and is less frequent among elite endurance athletes, and is indicative of decreased mitochondrial capacity of this haplogroup [30]. Patient P5, the least severely affected, belonged to haplogroup H. Mitochondrial protein synthesis levels were higher in cybrids from haplogroup H compared to haplogroups U (of which haplogroup K is a subclade) [31] and J [32]. Thus haplogroup H may offer some protection against the effects of the *YARS2* mutation which causes reduced mitochondrial protein synthesis. The remaining 3 patients, who also showed differences in disease severity, were all haplogroup K which suggests that there may be additional factors that affect disease severity. All patients were Australian-born of Lebanese origin from four unrelated families consistent with p.Phe52Leu being a founder mutation [16]. Recently, a p.Gly46Asp *YARS2* mutation was reported to cause similar clinical features, ie muscle-specific RC deficiencies of complexes I, III & IV in a MLASA patient, also of Lebanese origin [33]. Hence patients of Lebanese origin presenting with MLASA symptoms at any age should be screened for *YARS2* mutations.

A patient with anaemia within the first year of life and mild lactic acidosis and myopathy was found to be compound heterozygous for two novel *YARS2* mutations, p.Gly191Asp and p.Arg360X. Gly191 is located within the catalytic domain of YARS2, just before cluster 1, which is known to interact with base-pair 1–72 of the tRNA^Tyr^ acceptor stem [24]. A mutation at this location may disturb positioning of the α-helix of cluster 1, and hence adversely affect the aminoacylation reaction. This was confirmed by *in vitro* aminoacylation assay of a recombinant p.Gly191Asp YARS2, which displayed a 38-fold loss in catalytic efficiency compared to the wild-type enzyme, and is predicted to result in reduced mitochondrial protein synthesis, as was seen with the p.Phe52Leu mutation [16]. The mRNA transcript produced from the c.1078C > T (p.Arg360X) *YARS2* mutant was unstable and is most likely degraded by nonsense mediated decay due to the proximity of the mutation to an intron/exon boundary. There was no evidence of a truncated p.R360X YARS2 protein using an antibody targeted to the N-terminus of YARS2. Patient P6 YARS2 protein levels were maintained however, this is the less functional p.Gly191Asp YARS2. We consider that the mitochondrial RC disorder in patient P6 is a result of the combined effect of the p.Gly191Asp and p.Arg360X YARS2 mutations.

We did not identify pathogenic *YARS2* or *PUS1* mutations in the remaining nine patients, none of who had all the clinical features seen in those with pathogenic YARS2 mutations (Additional file 1). Common features in patients with pathogenic *YARS2* mutations are enzyme deficiencies of complexes I, III and IV in muscle, sideroblastic anaemia (although onset and duration may vary between patients), lactic acidosis, and skeletal myopathy and/or cardiomyopathy. However, it is possible that *YARS2* mutations may be identified in RC disorder patients with alternate phenotypes in future, as our cohort was selected based on presence of anaemia.

The basis for the muscle-specific manifestation of RC complex deficiencies in YARS2 patients appears due to an increased requirement for OXPHOS components in muscle compared to fibroblasts or myoblasts. Upon myogenesis, mitochondrial proliferation occurs, resulting in increased levels of RC complexes and mtDNA/nDNA [25], as we observed in control myoblasts. However, YARS2 patient cells were unable to induce sufficient expression of RC complex I, III and IV levels with myogenesis. Levels of porin and mitofilin increased, suggesting mitochondrial proliferation occurs, however YARS2 patient cells are unable to synthesise sufficient levels of mitochondrially encoded subunits of the RC complexes due to the defective aminoacylation activity of the mutant YARS2. This defect impacts mtDNA proliferation with myogenesis, possibly due to reduced intramitochondrial ATP levels, as high ATP concentrations are required for mtDNA transcription [34]. mtDNA transcription is a prerequisite for mtDNA replication, and mtDNA transcription and translation are also closely linked [35].

Muscle mitochondrial content is established during differentiation and development, and then maintained or modified throughout life [36]. The level of mtDNA/nDNA in muscle from P2 was comparable to controls, suggesting that over time, mitochondrial proliferation may have occurred as a compensatory response to the mitochondrial RC deficiency. The increased levels of PGC1-α and TFAM observed in patient muscle compared to controls are consistent with this hypothesis. In addition, in recently performed transcription profiling of P2 muscle before and after clinical improvement, mRNA levels for TFAM and PGC1-α were significantly higher than controls when the patient was displaying severe clinical features and returned to normal upon clinical improvement (data not shown). The elevated TFAM and PGC1-α protein levels with myogenesis are consistent with initiation of mitochondrial proliferation and suggest pressure on mitochondrial proliferative pathways as a consequence of low mtDNA copy number. Hence, it is possible that the defective translation caused by *YARS2* mutations, which results in mitochondrial RC dysfunction and decreased ATP production, also affects mtDNA transcription and replication, compounding the RC defect.

In conclusion, we have found that a homozygous YARS2 p.Phe52Leu mutation is a frequent cause of MLASA among patients of Lebanese origin, with patients displaying both inter- and intra-familial phenotypic variability. We have also identified a patient compound heterozygous for two novel *YARS2* mutations (p.Gly191Asp and p.Arg360X). We refine the clinical hallmarks of *YARS2* pathology to include muscle-specific defects in complexes I, III and IV, with sideroblastic anaemia and skeletal myopathy and/or cardiomyopathy. The tissue-specific effects of YARS2 disorders are related to the higher requirement for OXPHOS components in muscle. Further studies, including transcriptomic studies, may help reveal the compensatory mechanisms that are activated in response to the YARS2-induced RC dysfunction.

## Abbreviations

ARS2: Mitochondrial aminoacyl-tRNA synthetase; MLASA: Myopathy, lactic acidosis, sideroblastic anaemia; RC: Respiratory chain; YARS2: Mitochondrial tyrosyl-tRNA synthetase.

## Competing interests

The authors declare that they have no competing of interest.

## Authors’ contributions

LR conceived experiments, performed mutation screening, cloning, immunoblotting, mtDNA/nDNA determination and wrote the paper. MM performed mutation screening, cloning and enzyme assays. JR performed the protein expression and aminoacylation assays and wrote the corresponding section of the paper. RD and MD provided the mtDNA sequencing and haplotype analysis. PL, AR and MT provided patient samples and clinical information. SC conceived experiments and was involved in writing the paper. JC conceived experiments, provided patient samples and clinical information. All authors read and approved the paper.

## Supplementary Material

Additional file 1 **Clinical features of patients who did not have a pathogenic *YARS2* mutation.** Clinical features are described for the patients who did not have a pathogenic *YARS2* mutation. Non-pathogenic *YARS2* variants were identified in patients 7 and 8. Aminoacylation data for the variants shows they only have mild effects and probably are not pathogenic. Click here for file

Additional file 2 **mtDNA variations in p.Phe52Leu YARS2 patients.** mtDNA variations and their effects are shown for each of the p.Phe52Leu YARS2 patients. Novel variations are highlighted in blue. Click here for file

Additional file 3 **Comparison of native and MyoD forced myogenesis.** Transdifferentiation of fibroblasts (Fib) to myotubes (Myo) using a MyoD lentiviral vector (Myo-D forced myogenesis) gives rise to similar RC complex and YARS2 expression levels as seen in native *in vitro* myogenesis, involving differentiation of myoblasts (Myb) to myotubes (Myt), in both control (C) and patient (P2) cell lines. Click here for file

Additional file 4 ***YARS2* mRNA levels in Patient P6.** Sequencing chromatograms of P6 gDNA (A) and cDNA (B) showing the heterozygous c.1078C > T (p.Arg360X) mutation. The mutant transcript is less abundant than the wild-type, demonstrated by the lower peak height of the mutant “T” in the cDNA (B) compared to the wild-type “C”. Total *YARS2* mRNA level in P6 was ~50% of control levels (C). *YARS2* mRNA level was determined by qPCR and normalised to mRNA level of a housekeeping gene, RPL13A. Results are presented as mean fold change relative to control +/- SD (n ≥ 4). Click here for file
