# Supplementary material for: Phenotypic variability and identification of novel YARS2 mutations in YARS2 mitochondrial myopathy, lactic acidosis and sideroblastic anaemia
Source: Orphanet J Rare Dis. 2013 Dec 17;8:193. doi: 10.1186/1750-1172-8-193 (PMC3878580; doi:10.1186/1750-1172-8-193)
Supplement: Additional file 1 — Clinical features of patients who did not have a pathogenic YARS2 mutation. Clinical features are described for the patients who did not have a pathogenic YARS2 mutation. Non-pathogenic YARS2 variants were identified in patients 7 and 8. Aminoacylation data for the variants shows they only have mild effects and probably are not pathogenic. [file 1750-1172-8-193-S1.docx]

Additional File 1

Two patients, Patient 7 and Patient 8, were found to carry non-pathogenic *YARS2* variations (Table S1).

Patient 7, the child of non-consanguineous parents of Italian ancestry, had aregenerative anemia from birth, requiring transfusion every 40 days. When reviewed at 2 yo he had persistent lactic acidosis (3-5 mmol/L) which was corrected with bicarbonate, and some hepatic cytolysis. Erythropoietin treatment was ineffective for the anaemia. On respiratory chain enzymology, a Complex I deficiency was identified in fibroblasts, lymphocytes and muscle. No mitochondrial DNA defects were found.

Patient 8, a boy of French ancestry developed sideroblastic anemia at 6 years of age, becoming transfusion dependent at around 17 years of age. He also developed chronic diarrhea of unknown etiology at 16 years of age.  At 28 years of age he was found to have muscle weakness and bilateral ptosis. At this time his weight was only 39 kg and height 1.7 m. Pancreatic exocrine insufficiency was identified at this time. At 31 years of age he developed a severe cardiomyopathy and was found to have hyperlactatemia. Histochemical analysis of skeletal muscle showed ragged red fibres, and on enzymology he had complex I, III and IV deficiency in both skeletal muscle and cultured skin fibroblasts.

*YARS2* sequencing revealed that Patient P7 was homozygous for c.572G>T (p.Gly191Val), a SNP with a reported minor allele frequency of 0.1232 (dbSNP, NCBI). Patient P8 had a heterozygous c.1271G>A (p.Arg424Gln) variation which is reported to have a minor allele frequency of 0.0116 in European Americans (ESP, evs.gs.washington.edu). No *YARS2* deletions were detected in P7 and P8 using a quantitative PCR based method (data not shown; methods available on request). For P7 each parent was heterozygous for the detected variant. Parental DNA was not available for P8.

Gly191 lies within the catalytic domain of YARS2 and Arg424 lies within the ribosomal S4-like domain of unknown function. The protein prediction program SIFT (<http://sift.jcvi.org/>) predicts the Gly191Val and Arg424Gln variations to be tolerated. Tyrosylation assays on recombinant YARS2 proteins showed that the pGly191Val variant had a 3.6-fold decrease in catalytic efficiency, whilst the p.Arg424Gln variant resulted in a 2.2-fold reduction, compared to wild-type YARS2 (Table S2). These results were consistent with the SIFT predictions of the severity of these variations on protein function.

The mild effects of these YARS2 variants on catalytic activity, and their frequency within normal populations, provides strong evidence that they are not pathogenic. In addition, the clinical features of patients P7 and P8 vary from the patients with pathogenic YARS2 mutations. P7 (homozygous p.Gly191Val) did not have combined RC enzyme deficiency, only a complex I deficiency in muscle, with sideroblastic anaemia and lactic acidosis, but no myopathy. Whole exome sequencing has been performed on P7 DNA to identify the causal mutation. P8 (heterozygous p.Arg424Gln) had RC deficiencies of complexes I, III & IV in both muscle and fibroblasts and had sideroblastic anaemia and myopathy but no reported lactic acidosis. With only one *YARS2* variant identified in P8, mitochondrial DNA analysis was performed revealing large mtDNA deletions (data not shown). Thus, we believe disease pathology in P8 is due primarily to the mitochondrial DNA deletions.

Eight other patients were found to be negative for *YARS2* mutations. Clinical features of these patients are presented in Table S3.

**Table S1: Clinical data for patients with non-pathogenic *YARS2* variants**

| **Proband** | **P7** | | **P8** | |
| --- | --- | --- | --- | --- |
| **YARS2 variant** | c.[572G>T]; [572G>T]  p.[G191V];[G191V] | | c.[1271G> A];[=]  p.[R424Q];[=] | |
| **RC Enzyme Activity*^a^*** | Muscle | Fibroblast | Muscle | Fibroblast |
| **Complex I/CS** | 63*^b^* | 165 | 18 | ND |
| **Complex II/CS** | ND | 172 | 345 | 85 |
| **Complex III/CS** | 142*^b^* | 271 | 24 | 20 |
| **Complex IV/CS** | 126*^b^* | 274 | 11 | 25 |
| **Citrate Synthase*^c^*** | ND | 100 |  |  |
| **Sideroblastic Anaemia** | Severe  Transfusion dependent from birth | | Severe  From 6 yo  Transfusion dependent from 17 yo | |
| **Lactic Acidosis** | Moderate  3-4.5 mmol/L | | None | |
| **Myopathy** | None | | Severe | |
| **Other Features** | Mental retardation  Liver cytolysis  Currently 9 yo | | mtDNA deletions  Diarrhea  Cardiomyopathy  Ptosis  Pancreatic insufficiency  Growth hormone deficiency  Currently 41 yo | |

*^a^* values expressed as % residual activity

*^b^* values expressed as % Complex/Complex II residual activity as CS was not determined

*^c^*citrate synthase activity was expressed relative to protein

ND = not determined

**Table S2: Kinetic parameters for tyrosylation of tRNA^Tyr^ by wild-type and variant YARS2 recombinant proteins**

| **YARS2 Variant** | **Km (μM)** | **kcat (min-1)** | **kcat /Km**  **(Efficiency)** | **Loss of Efficiency^a^**  **(Fold change)** |
| --- | --- | --- | --- | --- |
| WT | 1 | 34 | 34 | 1 |
| p.Gly191Val | 0.9 | 8.6 | 9.6 | 3.6 |
| p.Arg424Gln | 0.8 | 12.5 | 15.6 | 2.2 |

*^a^* Loss of efficiency is calculated relative to the wild-type YARS2

**Table S3: Clinical data for patients with no detected *YARS2* variants**

| **Proband** | **P9** | **P10** | **P11** | **P12** | **P13** | **P14** | **P15** |
| --- | --- | --- | --- | --- | --- | --- | --- |
| **RC Enzymes Fibroblasts** | CI deficiency | CII, CIII, CIV deficiency | Normal | ND | CIV deficiency | Normal | ND |
| **RC Enzymes**  **Muscle** | ND | ND | CI, CIII, CIV deficiency | CI, CII, CIII, CIV deficiency | ND | Normal | ND |
| **RC Enzymes**  **Liver** | Normal | CI deficiency | ND | Normal | CII deficiency | Normal | ND |
| **RC Enzymes**  **Heart** | ND | ND | ND | ND | ND | CV deficiency | ND |
| **RC Enzymes**  **Lymphoblasts** | ND | ND | ND | ND | ND | ND | CIV deficiency |
| **Anaemia** | Sideroblastic | Sideroblastic | Anaemia | Macrocytic | Anaemia | Neonatal | Anaemia |
| **Lactic Acidosis** | + | + | + | + | + | + | - |
| **Myopathy** | Hypotonia | - | - | - | - | Hypotonia | - |
| **Other Features** | Hypoparathyroidism  Hypoglycemia  Cataracts  MRI basal ganglia anomalies | Hypoglycemia  Liver cytolysis & enlargement | Intrauterine growth retardation | Hepatocellular & renal insufficiency | Neonatal liver insufficiency  Renal insufficiency  Iron overload in liver & kidney  Hemochromatosis | Hypertrophic cardiomyopathy  Hypocitrulinemia  Hyaline membrane disease  Jaundice  High L/P ratio | Intrauterine growth retardation  Encephalopathy |

ND = not determined
