# Supplementary figures and images for: Phenotypic variability and identification of novel YARS2 mutations in YARS2 mitochondrial myopathy, lactic acidosis and sideroblastic anaemia
Source: Orphanet J Rare Dis. 2013 Dec 17;8:193. doi: 10.1186/1750-1172-8-193 (PMC3878580; doi:10.1186/1750-1172-8-193)

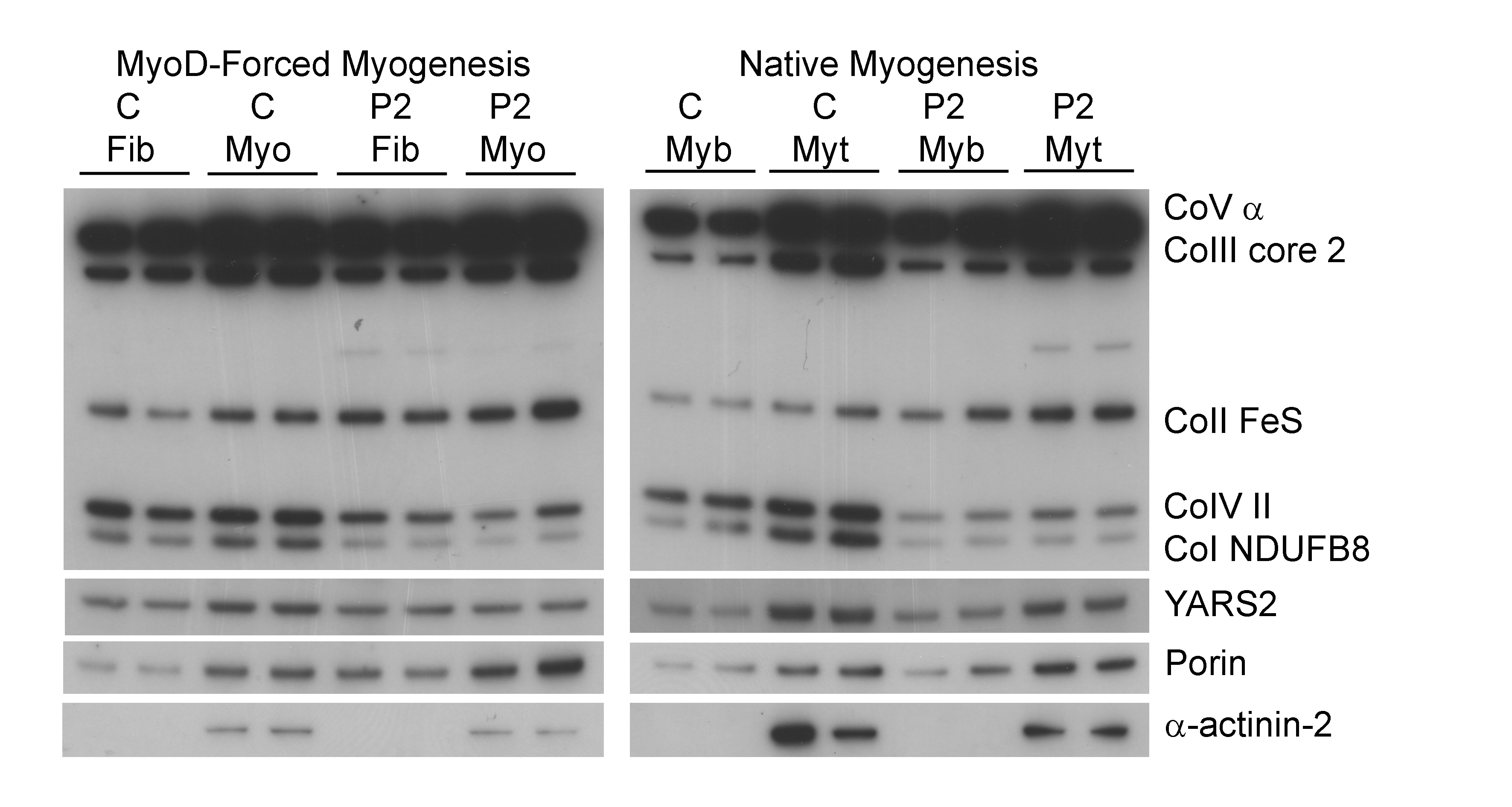

Supplement: Additional file 3 — Comparison of native and MyoD forced myogenesis. Transdifferentiation of fibroblasts (Fib) to myotubes (Myo) using a MyoD lentiviral vector (Myo-D forced myogenesis) gives rise to similar RC complex and YARS2 expression levels as seen in native in vitro myogenesis, involving differentiation of myoblasts (Myb) to myotubes (Myt), in both control (C) and patient (P2) cell lines. [file 1750-1172-8-193-S3.tiff]

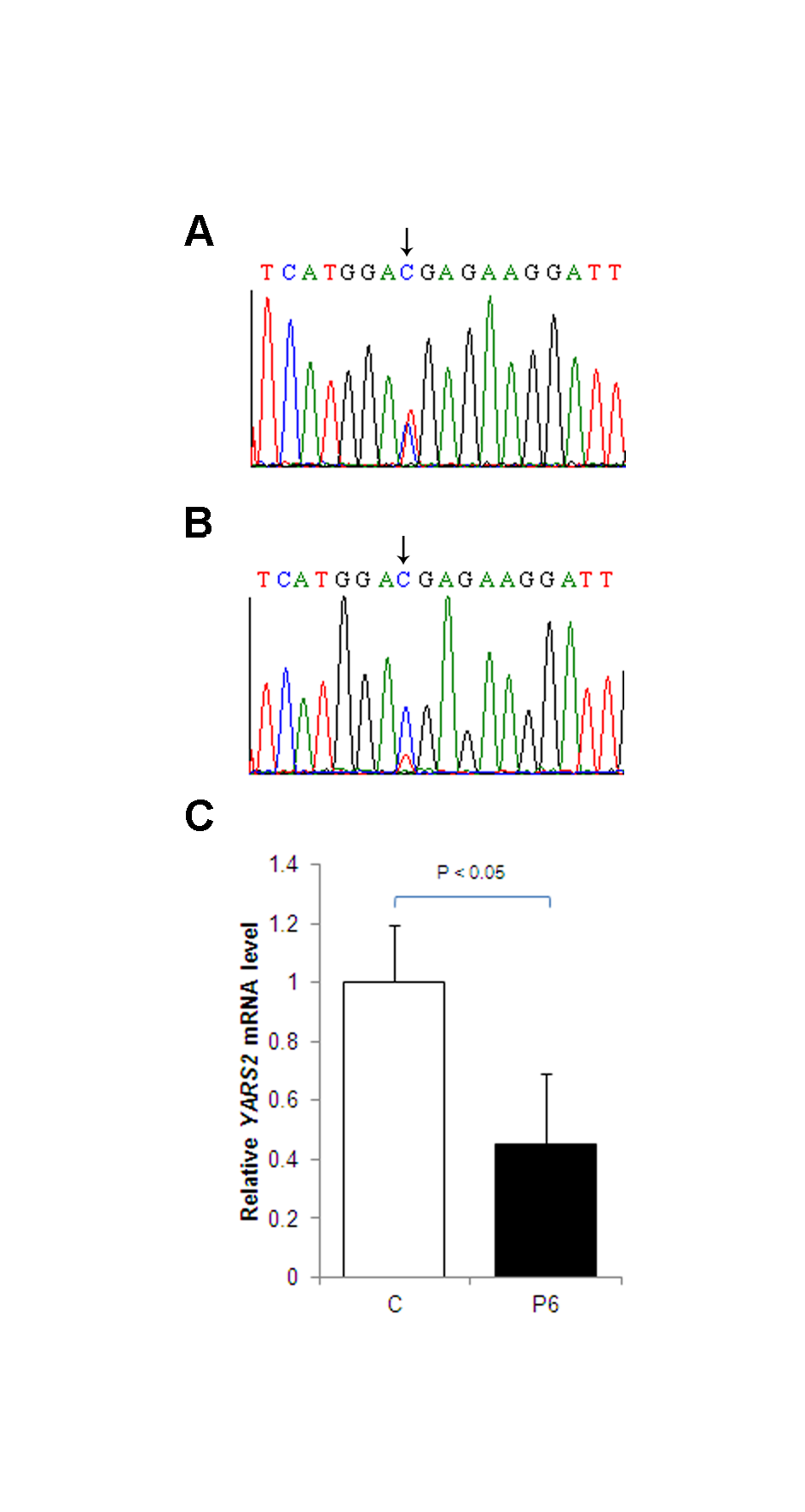

Supplement: Additional file 4 — YARS2 mRNA levels in Patient P6. Sequencing chromatograms of P6 gDNA (A) and cDNA (B) showing the heterozygous c.1078C > T (p.Arg360X) mutation. The mutant transcript is less abundant than the wild-type, demonstrated by the lower peak height of the mutant “T” in the cDNA (B) compared to the wild-type “C”. Total YARS2 mRNA level in P6 was ~50% of control levels (C). YARS2 mRNA level was determined by qPCR and normalised to mRNA level of a housekeeping gene, RPL13A. Results are presented as mean fold change relative to control +/- SD (n ≥ 4). [file 1750-1172-8-193-S4.tiff]
